# High-performance gene expression and knockout tools using sleeping beauty transposon system

**DOI:** 10.1186/s13100-018-0139-y

**Published:** 2018-11-26

**Authors:** Kaishun Hu, Yu Li, Wenjing Wu, Hengxing Chen, Zhen Chen, Yin Zhang, Yabin Guo, Yin Dong

**Affiliations:** 1 Guangdong Provincial Key Laboratory of Malignant Tumor Epigenetics and Gene Regulation, Medical Research Center, Sun Yat-Sen Memorial Hospital, Sun Yat-Sen University, Guangzhou, 510120 China; 2 Department of Breast Oncology, Sun Yat-Sen Memorial Hospital, Sun Yat-Sen University, Guangzhou, 510120 China

**Keywords:** Sleeping beauty, FBW7, CRISPR, NFATc1, RCC2, BRD7

## Abstract

**Background:**

Similar to retro−/lenti- virus system, DNA transposons are useful tools for stable expression of exogenous genes in mammalian cells. Sleeping Beauty (SB) transposon has adopted for integrating genes into host genomes in recent studies. However, SB-derived vector system for proteins purifying/tracking and gene knockout are still not available.

**Results:**

In this study, we generated a series of vectors (termed as pSB vectors) containing Sleeping Beauty IRDR-L/R that can be transposed by SB transposase. Gateway cassette was combined to the pSB vectors to facilitate the cloning. Vectors with various tags, Flag, Myc, HA, V5 and SFB, were generated for multiple options. Moreover, we incorporated the CRISPR-Cas9 cassette into the pSB plasmids for gene knockout. Indeed, using one of these vectors (pSB-SFB-GFP), we performed Tandem Affinity Purification and identified that NFATc1 is a novel binding partner of FBW7. We also knocked out RCC2 and BRD7 using pSB-CRISPR vector respectively, and revealed the novel roles of these two proteins in mitosis.

**Conclusion:**

Our study demonstrated that the pSB series vectors are convenient and powerful tools for gene overexpression and knockout in mammalian cells, providing a new alternative approach for molecular cell biology research.

**Electronic supplementary material:**

The online version of this article (10.1186/s13100-018-0139-y) contains supplementary material, which is available to authorized users.

## Background

Engineered gene expression is a basic technique in molecular and cellular biology investigations. Vectors containing exogenous genes can be transfected into mammalian cells by chemical transfection or electroporation. Unlike in bacteria or yeast cells, plasmids usually can’t be maintained permanently in mammalian cells. It takes long time to get stable expression of exogenous genes in cell lines using virus-free integrating vector, such as pcDNA3 series of vectors. To achieve stable expression, retro−/lenti- virus systems are the most popular options. However, the utility of retro−/lenti- viral vectors is heavily restricted by the size of genes. The efficiency of virus package drops dramatically when a large gene cloned into retro−/lenti- viral vector.

Transposon system is another option for the delivery of genes. Transposons, also known as transposable elements (TE) or jumping genes, comprise DNA transposons and retrotransposons. Neither transcription nor package is involved in the life cycle of DNA transposons, which makes transposon system simple and ideal tools for delivering genes, especially those larger ones. Sleeping Beauty (SB) transposon, a member of the Tc1/mariner family, is originally synthesized according to the consensus sequences from Salmonid fish [1]. The SB transposase has been optimized for higher efficiency in the consequent studies. SB100X, the latest version of SB transposase, has a highest transposition efficiency comparing to the earlier versions [2]. SB transposon is an important genetic tool in vertebrate system. Due to its high transposition efficiency and unbiased integration preference [3], SB is widely used for generating mutations systematically in both mice [4–6] and mammalian cells [7]. SB is also used in gene delivery of regular experiments [8–12], as well as in gene therapy [13–16].

In the current study, we developed a series of vectors with various gene expression cassettes flanked by SB inverted repeats, inverted repeat-direct repeat left/right regions (IRDR-L/R), which is recognized by the SB transposase, providing a great convenience tool for molecular cell biology experiments: a) CAG promoter was employed for high expression; b) Gateway design was combined with the vectors to make constructions more convenient; c) vectors with various tags, Flag, HA, GFP, etc., offer more options for different purposes; d) A system expresses N-terminally triple-tagged SFB (S-protein, Flag, and streptavidin-binding peptide) peptides for tandem affinity purification; e) SB delivered CRISPR-Cas9 system was also created to achieve virus-free gene knockout.

## Results

### The construction of the SB delivered vectors: pSB system

**Fig. 1:**
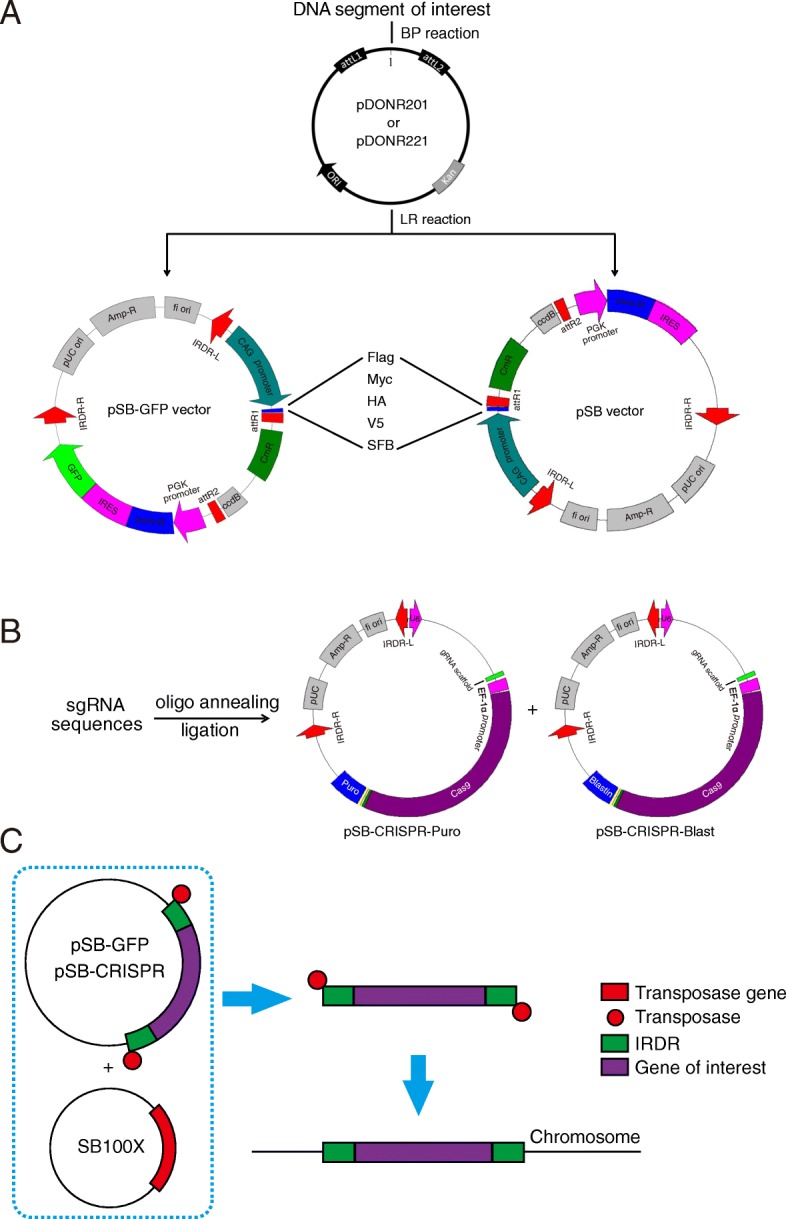
Overview of the Sleeping Beauty (SB) transposon system. **a** The Gateway cloning of pSB vectors. cDNA is cloned into an Entry vector between the attL1 and attL2 sites. In the presence of the LR clonase, recombination occurs between attL1-attR1 and attL2-attR2 to transfer the insert from the Entry vector into the Destination vector of choice. All Entry vectors contain the kanamycin resistance gene whereas all Destination vectors carry the ampicillin resistance gene. **b** sgRNA can be expressed in either pSB-CRISPR-Puro or pSB-CRISPR-Blast vector for protein depletion. **c** The workflow for using the Sleeping Beauty transposon system. Puro, puromycin; Blast, blasticidin

The vectors for gene overexpression were derived from a vector described previously [7]. In brief, the vector contains CAG promoter, V5 tag, Gateway cassette and PuroR-IRES-GFP, and the elements above are flanked by Sleeping Beauty inverted repeats (IRDR-L/R). We substituted the V5 tag with different tags (Myc, Flag, HA and SFB), resulting in a series of vectors with various tags. For each of them, we also made two versions, with or without GFP. Overall, ten overexpression vectors were constructed and termed pSB plasmids (Fig. 1a).

The vectors for gene knockout (pSB-CRISPR) were derived from the CRISPRv2 vector (addgene plasmid # 52961) [17]. The U6-sgRNA scaffold-Cas9-PuroR cassette of CRISPRv2 was amplified and inserted between the IRDR-L/R of pSB plasmid (with the gene expression cassette removed). Besides the puromycin resistant version (pSB-CRISPR-Puro), we also created a blasticidin resistant version (pSB-CRISPR-Blast), which can be transfected together with the pSB-CRISPR-Puro vector and selected with puromycin and blasticidin simultaneously (Fig. 1b). When co-transfected with SB100X plasmid (addgene plasmid # 34879) [2], the cassette between the SB IRDR-L/R will be cleaved and integrated into chromosomes of the host cell, causing exogenous gene overexpression or endogenous gene knockout stably (Fig. 1c).

### pSB vectors were tested and a novel FBW7 associated protein was identified through tandem affinity purification using pSB-SFB vector

**Fig. 2:**
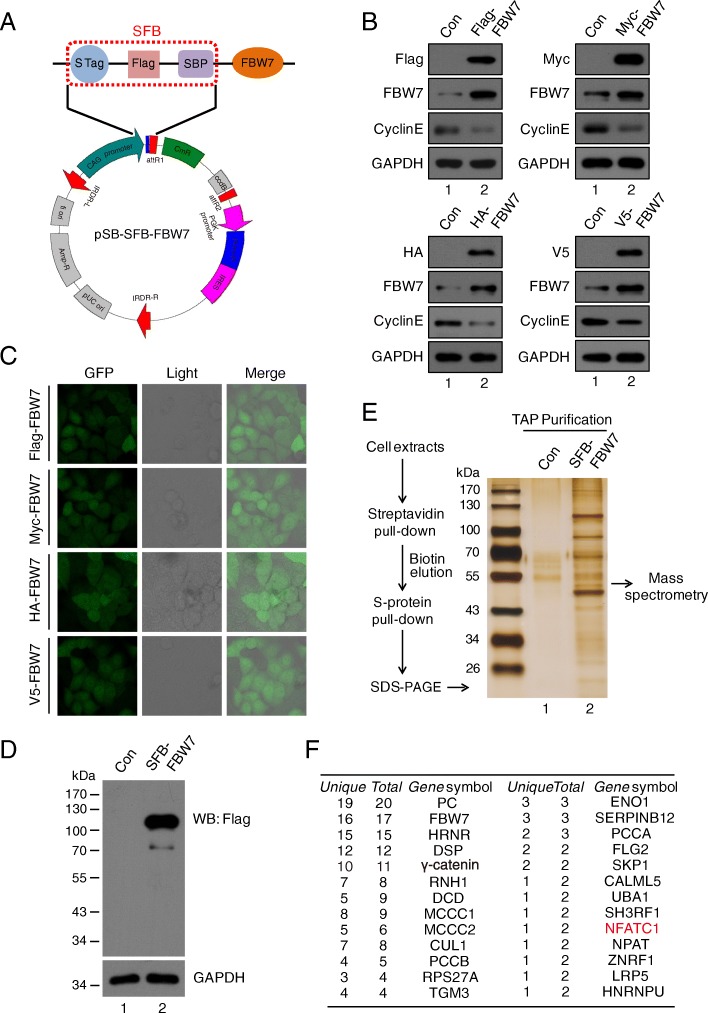
Overexpression of FBW7 using various vectors and identification of new associated protein of FBW7 though Tandem Affinity Purification. **a** Map of pSB-SFB-FBW7 vector. SFB-tagged FBW7 protein is composed of S-Peptide, Flag, and Streptavidin-binding Peptide. **b** The expression of FBW7 and its downstream target cyclin E in HeLa cells were analyzed using Western blot with indicated antibodies. **c** Four HeLa cell lines expressing either Flag-FBW7, Myc-FBW7, HA-FBW7 or V5-FBW7 are indicated. HeLa cells were co-transfected with pSB-GFP-Flag/Myc/HA/V5-FBW7 and SB100X plasmids for 24 h, and then selected with puromycin for 72 h. **d** HeLa cells stably overexpressing SFB-FBW7 fusion protein analyzed by Western blot (*n* = 3). **e** Silver staining of the SFB-FBW7 complex in SDS-PAGE gel. The whole-cell extracts were prepared from HeLa cells, and the purification steps were as indicated (n = 3). **f** Identification of FBW7 interacting protein by mass spectrometry. FBW7-interacting proteins, including FBW7 and NFATc1, are indicated (n = 3)

To evaluate the efficiency and feasibility of the Sleeping Beauty transposon system (Fig. 1a and 2a), we used FBW7 as an example to monitor protein expression in live cells. FBW7 is an F-box protein that recruits substrates for the SCF^FBW7^ E3 ubiquitin ligase. SCF^FBW7^ degrades several well-known oncoproteins, including Cyclin E [18], Notch [19], c-Jun [20] and c-Myc [21]. FBW7 has been demonstrated to play important roles in various physiological and pathological processes, such as tumorigenesis, cell proliferation, stemness and differentiation [22]. After FBW7 coding region subcloned into pSB vectors, GFP signal can be easily detected by fluorescence microscope (Fig. 2c). Furthermore, as shown in Fig. 2b, the expression of FBW7 increased up to 3–5 folds compared with the control groups, whereas the target gene Cyclin E was declined significantly, demonstrating that the Sleeping Beauty transposon system has high efficiency for intergrating genes into host genome. We next evaluated a system developed for tandem affinity purification which expresses N-terminally triple-tagged (S-protein, Flag, and streptavidin-binding peptide) proteins to see if it has good advantages in purifying protein (Fig. 2a). HeLa cells was stably transfected and expressed SFB-FBW7 (Fig. 2d). After a tandem affinity purification (TAP) scheme, proteins associated with FBW7 were identified by silver staining following by mass spectrometry analysis (Fig. 2e and f). Besides the known FBW7-binding proteins, such as Cul1, SKP1 [22], we also identified NFATc1 (nuclear factor of activated T-cells, cytoplasmic 1) as a novel binding partner for FBW7 (Fig. 2f).

**Fig. 3:**
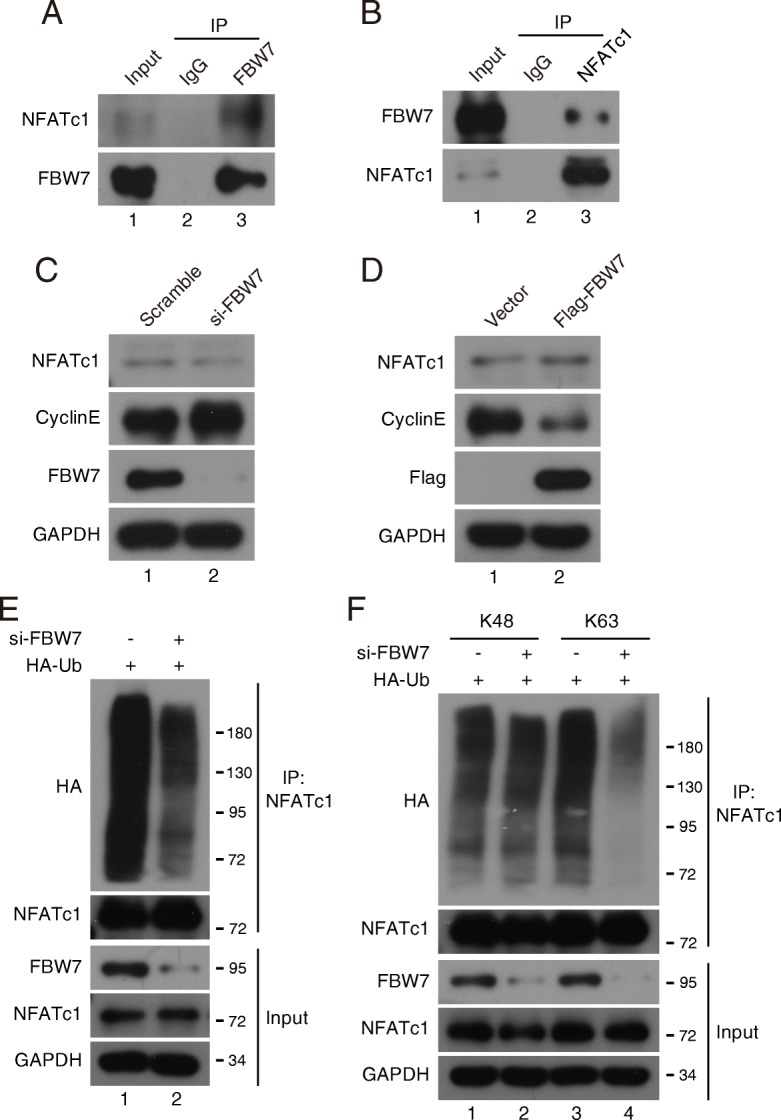
FBW7 interacts with NFATc1 and promotes its Lys63-linked polyubiquitylation. **a** and **b** HeLa cells were subjected to immunoprecipitation using anti-IgG, anti-FBW7 or anti-NFATc1 as indicated, and were analyzed by Western blot according to standard procedures (n = 3). **c** FBW7 was depleted in HeLa cells using FBW7 siRNA. The depletion of FBW7 did not affect NFATc1 protein stability (n = 3). **d** Transfection of FBW7-expressing plasmid in HeLa cells for 24 h and then analyzed by Western blot (n = 3). **e** Wild-type and FBW7-depleted HeLa cells were transfected with HA-Ub plasmids for 24 h and MG132 was added for another 4 h, then the cell lysates were subjected to IP using an anti-NFATc1 antibody followed by Western blot analysis (n = 3). **f** Wild-type and FBW7-depleted HeLa cells were transfected with HA-Ub (Lys48, Lys63 only) as indicated and immunoblotted with anti-HA antibody to detect ubiquitylated NFATc1 (n = 3)

To further confirm the high efficiency and fidelity of pSB-SFB vector in identifying potential interacting protein, we performed immunoprecipitation and in vivo ubiquitination assay to validate whether FBW7 is associated with NFATc1 and modulates its transcriptional activity. NFATc1 is a transcription factor that involved in T-cell development, osteoclastogenesis, and macrophage function [23–25]. However, recent studies have begun to characterize its roles in tumor cells. Activation of NFATc1 induces transcription of the c-myc gene and thereby promotes cell proliferation and anchorage-independent growth in pancreatic cancer cells, indicating that NFATc1 may play a vital role in carcinogenesis [26]. Oikawa et al. showed that NFATc1 induces expression of the transcriptional repressors Snail and Zeb1, resulting in downregulation of E-cadherin expression and changing cell morphology [27]. Of note, the phosphorylation of NFATc1 by DYRK1a enhanced NFATc1 protein stability by reducing its ubiquitination [28]. However, the detailed mechanism of regulating NFATc1 transcriptional activity remains unclear. Using TAP purification system combined with mass spectrometry, we identified NFATc1 as a potential substrate of FBW7 (Fig. 2e and f). Co-immunoprecipitation detected the interaction between FBW7 and NFATc1 at endogenous levels (Fig. 3a and b). We tested whether FBW7 might affect NFATc1 protein stability, and found that depletion of FBW7 did not alter the protein level of NFATc1, whereas depletion of FBW7 increased the protein expression of cyclin E, consistant with previous report [18] (Fig. 3c). Overexpression of FBW7 also had little effect on the protein stability of NFATc1 in HeLa cells (Fig. 3d), indicating that the binding of FBW7 to NFATc1 did not promote its degradation. Because FBW7 is a substrate recognition component of the SCF E3 ubiquitin ligase, we next examined whether FBW7 promotes NFATc1 ubiquitylation. As shown in Fig. 3e, the polyubiquitinated NFATc1 was decreased in FBW7-depleted HeLa cells. These results indicated that FBW7-mediated ubiquitylation of NFATc1 may affect on its function, but not on its stability.

In most cases, FBW7 recognizes and binds its substrates, followed by targeted ubiquitylation and subsequent degradation [22]. However, recent studies have shown that non-proteolytic ubiquitylation mediated by FBW7 plays a crucial role in the DNA damage response, which is mediated by K63-linker ubiquitylation [29, 30]. In general, polyubiquitylation via K48 commits the substrate to degradation by the 26S proteasome, whereas monoubiquitylation or K63-linked polyubiquitylation specifies nonproteolytic fates for the substrate [31]. To elucidate the styles of Ub chain linkages of NFATc1, we performed in vivo ubiquitination assay and found that K63-linked polyubiquitylation of NFATc1 was decreased in FBW7-depleted cells, whereas little change occurred at K48-linked polyubiquitylation in FBW7-depleted cells (Fig. 3f). Together, these results demonstrate that FBW7 interacts with NFATc1 to promote its polyubiquitylation via the K63 linkage, which may affect NFATc1 function. More importantly, these data indicated that combining the pSB-Flag/Myc/HA/V5 vectors with pSB-SFB vector is a very convenient and highly efficient platform to achieve stable expression of target genes and identify novel protein interactors.

### The high efficiency and fidelity of pSB-CRISPR vector in validating the role of RCC2 in mitotic entry and exit

**Fig. 4:**
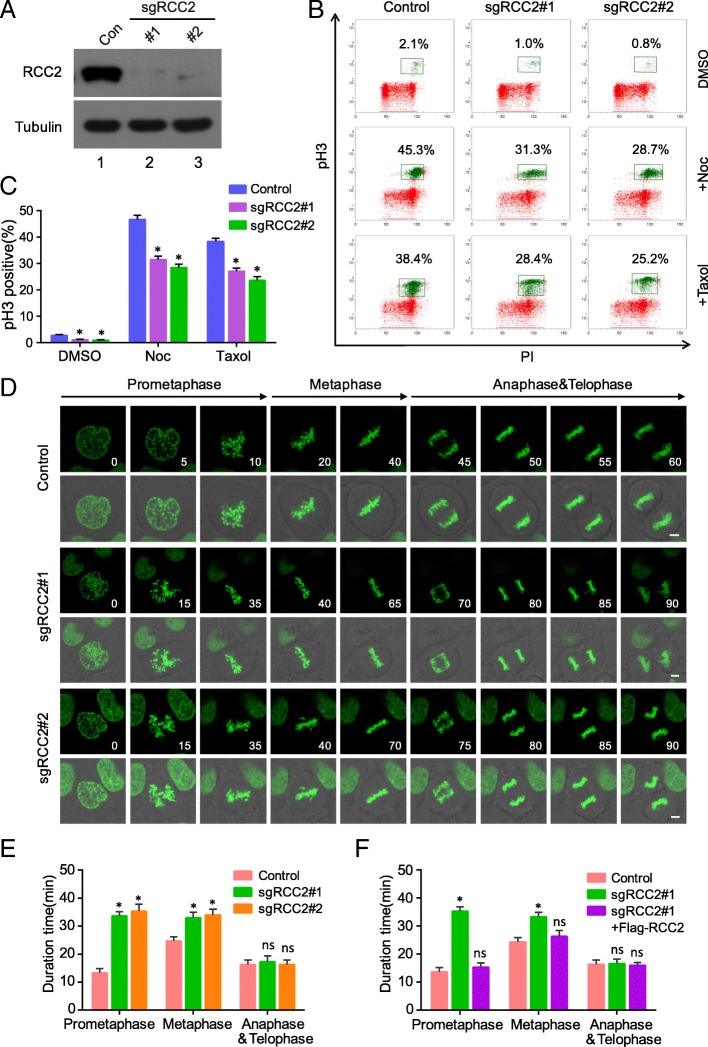
Depletion of RCC2 impairs mitotic entry and prometaphase to metaphase progression. **a** RCC2 was knocked out in HeLa cells by CRISPR, and Western blot was performed with indicated antibodies (n = 3). **b** Wild-type and RCC2-depleted HeLa cells were incubated with Nocodazole (100 ng/ml) or Taxol (2 μM) for 12 h, harvested and analyzed using flow cytometry (n = 3). The percentage of mitotic cells positive for phospho-histone H3 (pH 3) is indicated. **c** Quantitative results representing the mean ± SD of three independent experiments. Error bars indicate SD. *, *p* < 0.05. PI, propidium iodide. **d** Time-lapse images showing prolonged prometaphase and metaphase progression in RCC2-depleted HeLa-H2B cells, compared with control. Scale bars, 2 μm; the unit for time is minute. **e** Quantification of mitotic cells showed in D (*n* = 15 mitotic cells per group) and results represent mean ± SD. Error bars indicate SD. *, p < 0.05. (F) A RCC2-deficient HeLa cell line stably expressing Flag-tagged RCC2 was generated. Quantification of mitotic cells showed in Additional file 1: Figure S1C (n = 15 mitotic cells per group) and results represent mean ± SD. Error bars indicate SD. *, p < 0.05. NS, not significant

To determine the efficiency and feasibility of pSB-CRISPR vector in gene functional study, we took RCC2 as an example to validate its roles in mitosis. RCC2, also known as TD-60, was originally identified using human autoimmune antiserum at the anaphase spindle midzone [32]. RCC2 have been identified as a component of the chromosomal passenger complex (CPC) combined with Aurora B kinase [33], INCENP [34] and Survivin [35], involving in chromosomes and spindle assembly and mitotic exit. First, we constructed two specific guide RNA (sgRNA) targeting human RCC2 gene into pSB-CRISPR vector, and endogenous RCC2 were completely suppressed in both sgRNA-treated HeLa cells (Fig. 4a). Next, we selected two anti-mitotic drugs usually used for mitotic arrest: nocodazole, a rapidly-reversible inhibitor of microtubule polymerization, which blocks cells in prometaphase [36]; and Taxol (paclitaxel), an irreversible stabilizer of microtubule polymer, which blocks cell cycle progress at the metaphase/anaphase transition (Additional file 1: Figure S1A) [37]. Phospho-ser10-histone3 (pH 3), an indicator of cells at M phase, was used to monitor the G2-M transition in HeLa cells. Indeed, as shown in Fig. 4b and c, the percentage of cells at M phase was remarkably decreased by depletion of RCC2, suggesting that RCC2 is important for G2-M progression. This is similar to the report of Mythili Y et al, showing that RCC2 is required for progression of G2 cells into mitosis [38]. To further examine the role of RCC2 during mitotic progression, we integrated GFP-H2B into HeLa cells to monitor mitotic progression. As shown in Fig. 4d and e, depletion of RCC2 significantly delayed mitotic progression from prometaphase to metaphase, whereas, there was little effect on anaphase to telophase progression in RCC2-depleted cells, which is also in consistence with previous report [39]. Furthermore, this delayed mitotic progression in RCC2-depleted cells were rescued in cells stably expressing Flag-tagged RCC2 (Fig. 4f, Additional file 1: Figure S1B and S1C), ruling out the possibility that our observations were due to sgRNA off-target effects. Collectively, these findings demonstrate that RCC2 is an essential regulator of cell cycle progression during G2-M transition and mitosis, and pSB-CRISPR vectors are powerful tools in gene function study.

### Exploring novel function of BRD7 in mitosis using pSB-CRISPR vector

**Fig. 5:**
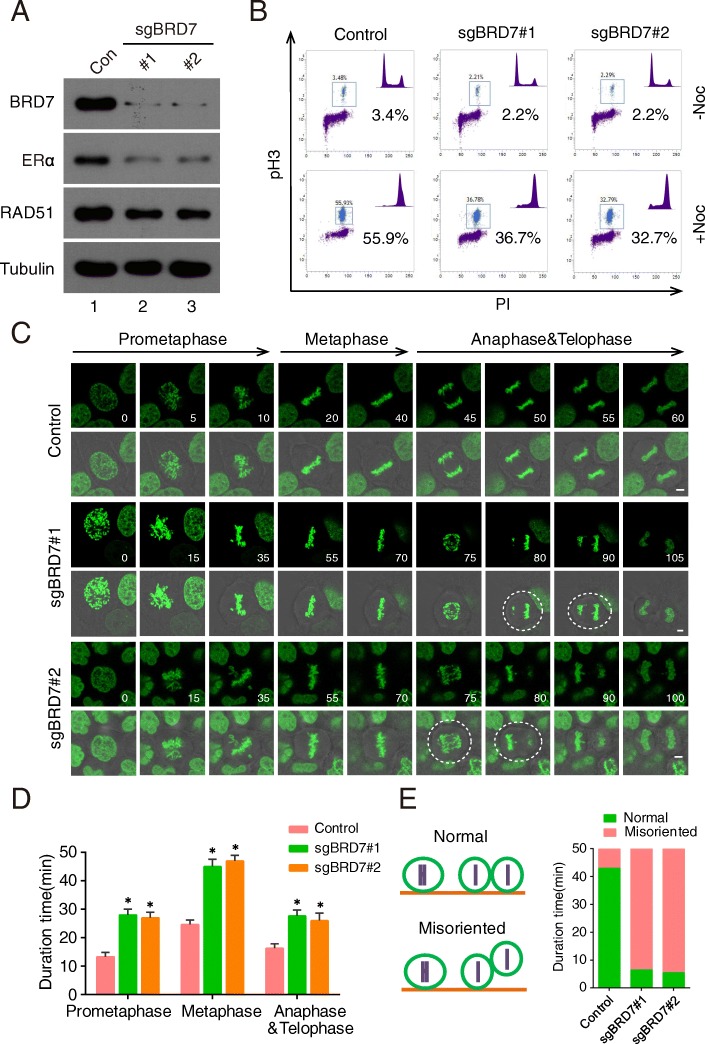
BRD7 is important for oriented cell division. **a** Immunoblots for BRD7 and target gene expression (ERα and RAD51) in control and BRD7-depleted HeLa cells (n = 3). **b** Wild-type and BRD7-depleted HeLa cells were incubated with nocodazole (100 ng/ml) for 16 h, harvested and analyzed using flow cytometry. The percentage of cells positive for pH 3 is indicated (n = 3). **c** Time-lapse images showing prolonged prometaphase and telophase and misoriented cell division (uneven timing of daughter-cell adhesion to the substratum) in BRD7-depleted HeLa-H2B cells, compared with control. Dashed lines indicate misoriented cell divisions. (Scale bars, 2 μm). **d** Quantification of mitotic cells showed in C (n = 15 mitotic cells per group) and results represent mean ± SD. Error bars indicate SD. *, p < 0.05. **e** Quantification of normal and misoriented cell divisions in cells treated as in C (n = 15 mitotic cells per group). Values are mean ± SD. *, p < 0.05. NS, not significant

The advantage of Sleeping Beauty transposon system is for large genes delivering and no integration preference, which avoids the integrations into active genes or their promoter regions and has lower off-target effects [3, 10]. In our previous study, we found that BRD7 forms a complex with anaphase-promoting complex/cyclosome (APC/C) and is degraded by APC/C^cdh1^ and APC/C^cdc20^ during the cell cycle [40], indicating that BRD7 may play a pivotal role in mitosis. In this study, we engineered pSB-CRISPR vector to investigate the roles of BRD7 in mitosis successfully. First, endogenous BRD7 was effectively depleted using pSB-CRISPR vector with two specific sgRNAs, and its downstream genes, ERα and RAD51, were also decreased (Fig. 5a). The percentage of cells at M phase was remarkably decreased in BRD7-depleted cells compared with control group, suggesting that BRD7 is important for mitotic entry (Fig. 5b). To investigate the role of BRD7 in mitotic exit, BRD7 wildtype and BRD7-depleted cells were synchronized in M phase using nocodazole and then released. The mitotic exit progression in these cells was monitored, and mitotic exit was significantly delayed in cells depleted of BRD7 (Additional file 1: Figure 2A). Furthermore, time-lapse microscopy of HeLa cells stably expressing GFP-tagged histone 2B (GFP-H2B) revealed that depletion of BRD7 caused an obvious delay in mitotic exit due to the prolongation of prometaphase-metaphase-anaphase-telophase progression (Fig. 5c and d). In addition, BRD7-depleted cells displayed uneven timing of daughter cell adhesion to the substratum, suggesting that BRD7 might be required for proper orientation and positioning of the mitotic spindle (Fig. 5e, Additional file 1: Figure 2B).

**Fig. 6:**
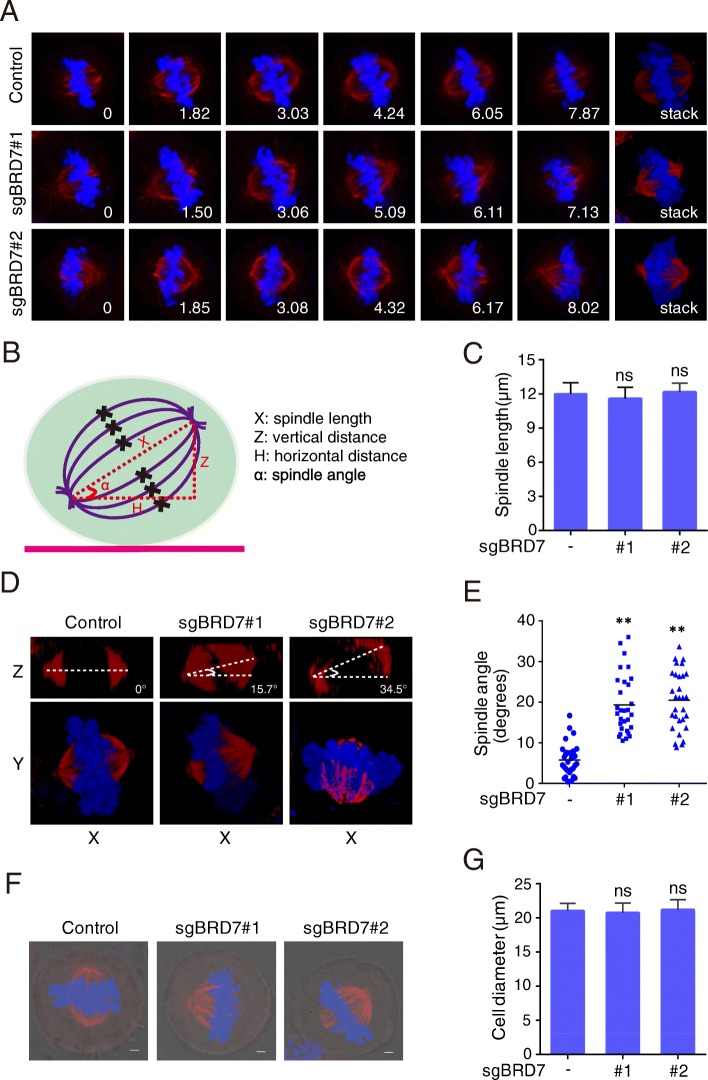
Depletion of BRD7 leads to spindle misorientation. **a** Cells were transfected with control or BRD7 sgRNAs and stained with anti-β-tubulin antibody (red) and DAPI (blue) and the image series of mitotic cells were shown. The position of the Z stage of the mitotic spindle is indicated in μm, and stack refers to the projected image. **b** Scheme for analysis of various parameters of the mitotic spindle: spindle angle (α) and spindle length (μm). (D) XY and XZ projections of confocal Z stacks taken of metaphase cells stained for β-tubulin (red) and DNA (blue). **c** and **e** Experiments were performed as in A, and the spindle length, the spindle angle between the two spindle poles were measured as described in B. **f** and **g** Immunofluorescence/phase-contrast images and cell diameter of control and BRD7-depleted metaphase HeLa cells stained with anti-β-tubulin antibody (red) and DNA (blue). *n* = 30 cells per group. Values are mean ± SD. *, p < 0.05, **, *p* < 0.01. NS, not significant. Scale bars, 2 μm

To clarify further the role of BRD7 in spindle orientation and positioning, we analyzed the Z-stage stacks of the mitotic spindle by confocal microscopy and measured various parameters of the mitotic spindle (Fig. 6a and b). We found that depletion of BRD7 expression did not obviously affect the spindle length (Fig. 6c). Immunofluorescence microscopy of fixed cells showed that BRD7 depletion greatly broadened the distribution of the spindle angles (Fig. 6d and e). The average spindle angle in BRD7-depleted cells was above 20 degrees, indicative of spindle misorientation, whereas the average spindle angle was less than 10 degrees in control cells. However, the loss of BRD7 did not obviously affect the gross morphology and cell diameter (Fig. 6f and g). Therefore, pSB-CRISPR vector system is an attractive solution that allows for easy and highly efficient gene knockout.

## Discussion

In this report, we have developed a series of vectors using Sleeping Beauty transposon system, providing alternative powerful tools for molecular cell biology study. These vectors can be efficiently and conveniently used in: 1) overexpressing target genes with different tags (Figs. 1 and 2); 2) purifying protein associators for mass spectrometry (Fig. 2); 3) delivering CRISPR-Cas9 system to achieve virus-free knockout (Figs. 4 and 5). Using this system, a novel FBW7-associated protein, NFATc1, was easily identified (Fig. 3). Moreover, mitosis-related factors: RCC2 and BRD7, were efficiently depleted in HeLa cells, further uncovering their roles in mitotic entry and exit (Figs. 4, 5 and 6).

We developed a series of vectors with great advantages by using Sleeping Beauty transposon system. First, pSB vectors are very easy to clone with Gateway reaction. Second, the high integration frequency of SB100X transposase and the strong CAG promoter guarantee high expression efficiency of target genes. Third, compared to virus vectors, there’s no limit for target gene and no safety concern of potential infection. Furthermore, we combined CRISPR-Cas9 with transposon system and achieved virus-free CRISPR knockout.

In this study, we show that NFATc1 is a novel substrate of FBW7, which promotes its lys63-linked polyubiquitylation (Fig. 3). NFATc1, belongs to transcription factors of the nuclear factor of activated T cells (NFAT) family, play key roles in inflammatory and immune responses [41]. Recently, studies have begun to uncover roles of NFATc1 in tumorigenesis. NFATc1 is highly expressed in aggressive cancer cells and tissues, and promotes invasion through the transcriptional induction of Snail and Zeb1 in a TGF-β independent manner [27, 42, 43]. Moreover, FBW7 has three isoforms (α, β, γ) that vary only in their N-terminal region and, consequently, their subcelluar localization (nucleus, cytoplasm, and nucleolus, respectively), likely confers compartment-dependent substrate specificity [44]. Therefore, we speculated that FBW7-mediated lys63-linked polyubiquitylation of NFATc1 may have important effect on its roles in nuclear compartment.

Off-targeting is one of the major challenges for genome editing introduced by CRISPR/Cas9, which was resulted from tolerance of mismatches between guide RNAs and genomic loci carrying similar sequences, leading to genome editing at unexpected sites and genome instability [45–47]. A number of approaches have been developed to enhance the fidelity of CRISPR-Cas9 mediated genome editing, including shortening guide sequences [48], pairing double nicking [49], engineering Cas9 [50, 51] and limiting Cas9 exposure with doxycycline (DOX)-inducible Cas9 (DOX-iCas9) [52]. All of these efforts were aimed at decreasing the binding affinity between Cas9/sgRNA and off-target sites. Thus, as long as Cas9 and guide RNA are both present in cells, they will keep potential risk of editing off-target sites. In our study, we engineered SB to deliver CRISPR-Cas9 cassette to achieve constitutively expressed Cas9 which may also result in potential off-target effects and genome instability. In our future studies, we will develop inducible pSB-CRISPR constructs which exploit SB to deliver DOX-inducible spCas9 cassette (addgene #85400) [52] and sgRNA scaffold (addgene #52963) [17] respectively to provide a solution for easy genome editing with tight temporal control and minimal off-target effects.

## Conclusions

In summary, we have successfully established a highly efficient pSB vector platform, to provide quick and simple methods for gene overexpression, knockout, and protein purification, allowing validation large number of genes or genes with large size. (NOTE: The pSB vectors will be submitted to addgene, when this manuscript is accepted.)

## Materials and methods

### Cells culture and transfection

HeLa cells were obtained from ATCC and cultured in DMEM (Life Technologies) supplemented with 10% fetal bovine serum (Life Technologies) with 5% CO2 at 37 °C. Plasmid transfections were performed using Lipofectamine 2000 (Life Technologies). siRNA transfection was performed using Lipofectamine RNAiMAX reagent (Life Technologies) according to its protocol. 48 h post-transfection, cells were harvested and subjected to Western blot. The FBW7 siRNA sequences as follow:

GCATATGATTTTATGGTAA, was described previously [53].

### Plasmids construction

For the generation of pSB-Flag/Myc/HA-GFP series, we employed mutagenesis kit (Takara, Japan) to introduce different expression cassettes. The primers used for amplification as follows: pSB-Flag-F: GACTACAAGGACGACGATGACAAGGAATTG.

ATCACAAGTTTGTA; pSB-Flag-R: CATGGTGGCGACCGGTAGCG, pSB-Myc-F: GAACAAAAACTCATCTCAGAAGAGGATCTGGAATTGATCACAAGTTTGTA; pSB-Myc-R: CATGGTGGCGACCGGTAGCG; pSB-HA-F: TACCCATACGATGTTCCAGA.

TTACGCTGAATTGATCACAAGTTTGTA; pSB-HA-R: CATGGTGGCGACCGGTAG.

CG. For the generation of pSB-Flag/Myc/HA series, which its GFP tag is deleted, we used the plasmid above to amplify GFP-lacked DNA fractions, and the primers used for amplification as indicated: pSB-dGFP-F: TGAGTTTGGACAAACCACAAC; pSB-dGFP-R: GGTTGTGGCCATATTATCAT; For the generation of pSB-SFB-GFP vector, the SFB DNA sequences was inserted to pSB-V5-GFP vector using the ClonExpress II One Step Cloning Kit (Vazyme, China), and all mutations were verified by DNA sequencing. For the construction of pSB-Flag-FBW7 or RCC2, the coding region of FBW7 and RCC2 were firstly subcloned into pDONR221 (Invitrogen) as entry clones and were subsequently transferred to Gateway-compatible destination vector (pSB-Flag) for the expression of Flag-tagged fusion protein.

### CRISPR-Cas9 knockout

For CRISPR/Cas9 knockout of human RCC2 and BRD7 in HeLa cells, the following small guide RNAs (sgRNAs) were used: sgRCC2#1: TTGTGTCTGCAGCATGTGGG.

CGG; sgRCC2#2: TGCAGTAGCAGCAGCGGCGG; sgBRD7#1: AGGCAAGTCTAA.

TCTCACAGGGG; sgBRD7#2: GATCGTTTTTGTCTTCGAAGAGG. The gRNA sequences were cloned into the vector pSB-CRISPR. Cells were transfected with indicated pSB-CRISPR plasmids and transposase SB100X followed by extensive selection with 2 μg/ml puromycin. The efficiency of knockout was identified by Western blot with indicated antibodies.

### The establishment of stable cell lines and tandem affinity purification of SFB-tagged FBW7

HeLa cells were transfected with plasmids encoding SFB-FBW7 and transposase SB100X. The cell was selected by culturing in medium containing puromycin (1 μg/ml) and confirmed by immunoblot and immunostaining. For affinity purification, ten plates of HeLa cells stably expressing tagged proteins were lysed with NETN buffer (20 mM Tris-HCl (pH 8.0), 100 mM NaCl, 1 mM EDTA, and 0.5% Nonidet P-40) for 20 min. The supernatants were cleared at 15000 g to remove debris and then incubated with streptavidin-conjugated beads (Amersham Biosciences) for 12 h at 4 °C. The beads were washed three times with NETN buffer, and then bead-bound proteins were eluted with NETN buffer containing 1 mg/ml biotin (Sigma). The elutes were incubated with S-protein beads (Novagen) for 2 h at 4 °C. The beads were washed three times with NETN buffer and subjected to SDS-PAGE. Protein bands were excised and digested, and the peptides were analyzed by mass spectrometry.

### Western blot and immunoprecipitation

Cells were lysed in RIPA buffer (50 mM Tris-HCl [pH 8.0], 5 mM EDTA, 150 mM NaCl, and 0.5% Nonidet P-40 and a protease and phosphatase inhibitor cocktail (Bimake, China)), and the clarified lysates were resolved by SDS-PAGE and transferred to PVDF membranes for Western blot using ECL detection reagents (Beyotime, China). The immunoblots were processed according to standard procedures using primary antibodies directed to FBW7 (Bethyl, A301-720A), BRD7 (Bethyl, A302-304A), Cyclin E (CST, 20808), GAPDH (CST, 2118), HA (CST, 3724), Flag (CST, 14793), Myc (CST, 2278), V5 (CST, 13202), RCC2 (CST, 5104), RAD51 (CST, 8875), ERα (Santa Cruz, SC-514857), tubulin (Santa Cruz, SC-166729), NFATc1 (Abiocode, R2315–1). For immunoprecipitation, the supernatants were first incubated with S-protein agarose (Novagen) overnight at 4 °C, and the precipitates were washed three times with NETN buffer. To detect endogenous interaction, the clarified supernatants were first incubated with anti-FBW7 or NFATc1 antibody for two h and then protein G-agaroses (Thermo Fisher, 10004D) overnight. After washed three times with NETN buffer, the samples were collected and analyzed by Western blot.

### In vivo ubiquitination assay

This procedure was performed as previously described [54]. Briefly, HeLa cells were transfected with the indicated plasmids for 24 h and were treated with 10 μM MG132 for 6 h prior to harvesting. The cells were lysed in RIPA buffer with protease and phosphatase inhibitor cocktail (Bimake, China). Endogenous NFATc1 was immunoprecipitated using anti-NFATc1 antibody for 12 h at 4 °C. Polyubiquitinated NFATc1 was detected using an anti-HA antibody.

### Phospho-histone H3 staining

Cells were incubated with 100 ng/ml of nocodazole or Taxol (2.5 μm) for 14 h and were harvested and fixed in 70% ethanol at − 20 °C overnight. Then, cells were resuspended in 1 ml of 0.25% Triton X-100 in PBS, and rotated at 4 °C for 15 min. After the cells were centrifuged, the cell pellet was suspended in 100 ml of PBS containing 1% bovine serum albumin and 2 μg Phospho-Histone H3 (Ser10) [Alexa Fluor 488 conjugate, CST, 3465], and incubated for 2 h at room temperature. Then, the cells were rinsed with PBS containing 1% bovine serum albumin and stained with propidium iodide, and cellular fluorescence was measured using a FC-500 flow cytometer (Beckman Coulter).

### Fluorescence microscopy

Cells were fixed with colded-methanol for 15 min and blocked with 5% bovine serum albumin in phosphate-buffered saline. Cells were then incubated in succession with primary and secondary antibodies followed by staining with DAPI and examined with a ZEISS confocal microscope (ZEISS-800, Germany) equipped with ZENblue2.3 software. The spindle angle and spindle length were measured as described previously [55]. For time-lapse microscopy, cells were cultured in a 37 °C chamber, and mitotic progression was recorded with the confocal microscope as described [56].

## Additional file

Additional file 1: **Figure S1**. Re-expression of Flag-tagged RCC2 rescued sgRCC2-mediated delay of mitotic exit. A. HeLa cells expressing GFP-H2B were incubated with Nocodazole (100 ng/ml) or Taxol (2 μM) for 12 h, and analyzed using ZEISS710 confocal microscope. (Scale bars, 2 μm). B. Immunoblots for control cells, RCC2-depleted cells, and RCC2-depleted cells stably expressing the indicated constructs. C. Time-lapse images showing mitotic exit in HeLa-H2B cells indicated above. **Figure S2**. Depletion of BRD7 delays mitotic exit and leads to spindle misorientation in HeLa cells. A. HeLa cells transfected with indicated sgRNA and synchronized in M phase by incubation with 100 ng/ml nocodazole for 14 h. M phase cells selected by shake-off were released for the indicated time. B. Time-lapse images showing prolonged metaphase to anaphase and misoriented cell division (uneven timing of daughter-cell adhesion to the substratum) in BRD7-depleted HeLa-H2B cells, compared with control. (Scale bars, 2 μm). (DOCX 7450 kb)

## Abbreviations

Blast: Blasticidin
IRDR-L/R: Inverted repeat-direct repeat left/right
Noc: Nocodazole
Puro: Puromycin
SB: Sleeping Beauty
SFB: S-protein, Flag, and streptavidin-binding peptide
TAP: Tandem affinity purification
Taxol: Paclitaxel
