# Correction to: High-performance gene expression and knockout tools using sleeping beauty transposon system

**DOI:** 10.1186/s13100-019-0145-8

**Published:** 2019-01-09

**Authors:** Kaishun Hu, Yu Li, Wenjing Wu, Hengxing Chen, Zhen Chen, Yin Zhang, Yabin Guo, Dong Yin

**Affiliations:** 1 Guangdong Provincial Key Laboratory of Malignant Tumor Epigenetics and Gene Regulation, Medical Research Center, Sun Yat-Sen Memorial Hospital, Sun Yat-Sen University, Guangzhou, 510120 China; 2 Department of Breast Oncology, Sun Yat-Sen Memorial Hospital, Sun Yat-Sen University, Guangzhou, 510120 China

**Correction to: Mob DNA**

**https://doi.org/10.1186/s13100-018-0139-y**

The original article [1] contained an error whereby author Dong Yin’s name was mistakenly inverted. This error has now been corrected.
