# Supplementary material for: High-performance gene expression and knockout tools using sleeping beauty transposon system
Source: Mob DNA. 2018 Nov 26;9:33. doi: 10.1186/s13100-018-0139-y (PMC6260868; doi:10.1186/s13100-018-0139-y)
Supplement: Supplementary file 1 — Figure S1. Re-expression of Flag-tagged RCC2 rescued sgRCC2-mediated delay of mitotic exit. A. HeLa cells expressing GFP-H2B were incubated with Nocodazole (100 ng/ml) or Taxol (2 μM) for 12 h, and analyzed using ZEISS710 confocal microscope. (Scale bars, 2 μm). B. Immunoblots for control cells, RCC2-depleted cells, and RCC2-depleted cells stably expressing the indicated constructs. C. Time-lapse images showing mitotic exit in HeLa-H2B cells indicated above. Figure S2. Depletion of BRD7 delays mitotic exit and leads to spindle misorientation in HeLa cells. A. HeLa cells transfected with indicated sgRNA and synchronized in M phase by incubation with 100 ng/ml nocodazole for 14 h. M phase cells selected by shake-off were released for the indicated time. B. Time-lapse images showing prolonged metaphase to anaphase and misoriented cell division (uneven timing of daughter-cell adhesion to the substratum) in BRD7-depleted HeLa-H2B cells, compared with control. (Scale bars, 2 μm). (DOCX 7450 kb) [file 13100_2018_139_MOESM1_ESM.docx]

**Supplementary Figure S1. Re-expression of Flag-tagged RCC2 rescued sgRCC2-mediated delay of mitotic exit.** A. HeLa cells expressing GFP-H2B were incubated with Nocodazole (100 ng/ml) or Taxol (2 μM) for 12 h, and analyzed using ZEISS710 confocal microscope. (Scale bars, 2 μm). B. Immunoblots for control cells, RCC2-depleted cells, and RCC2-depleted cells stably expressing the indicated constructs. C. Time-lapse images showing mitotic exit in HeLa-H2B cells indicated above.


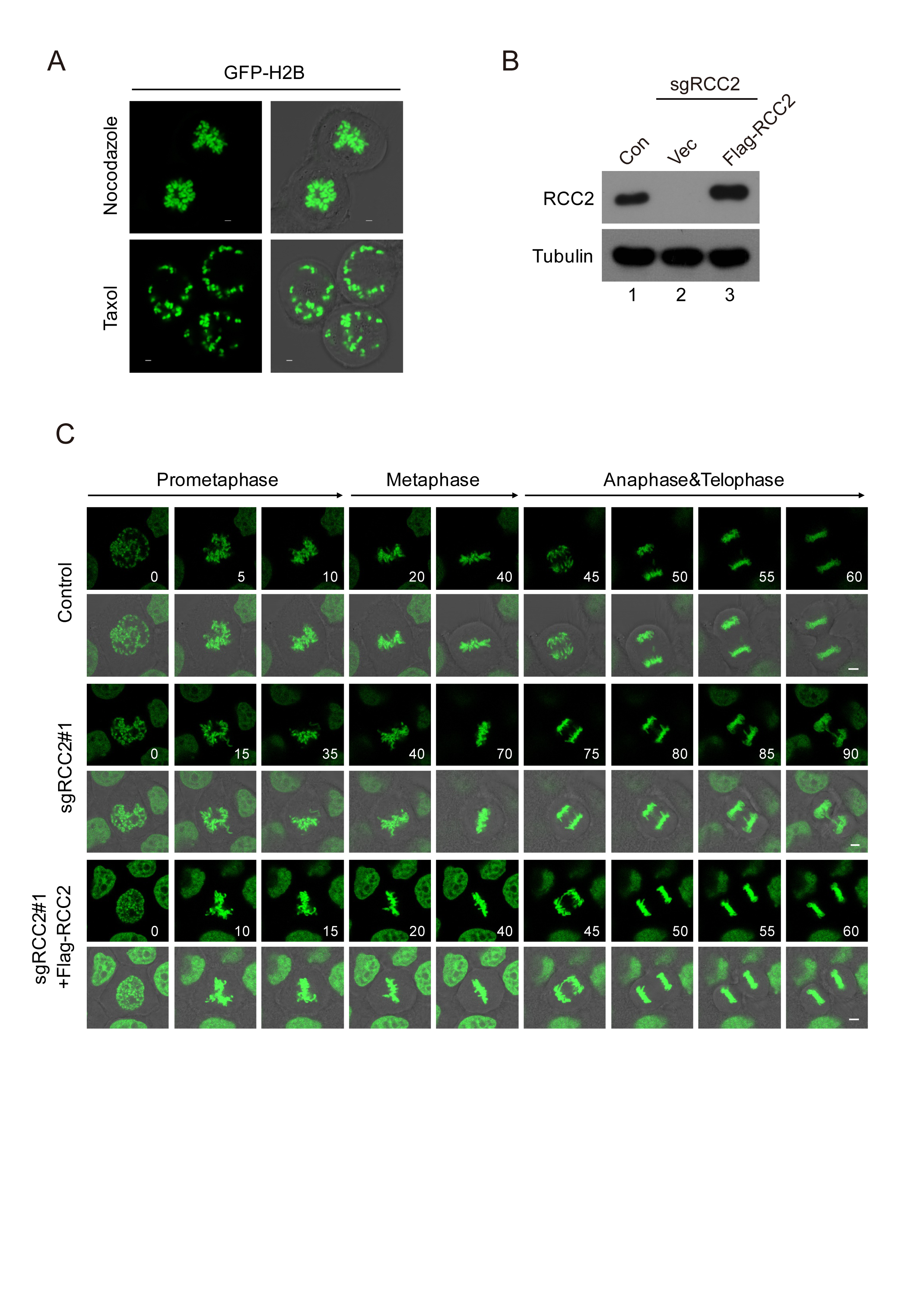

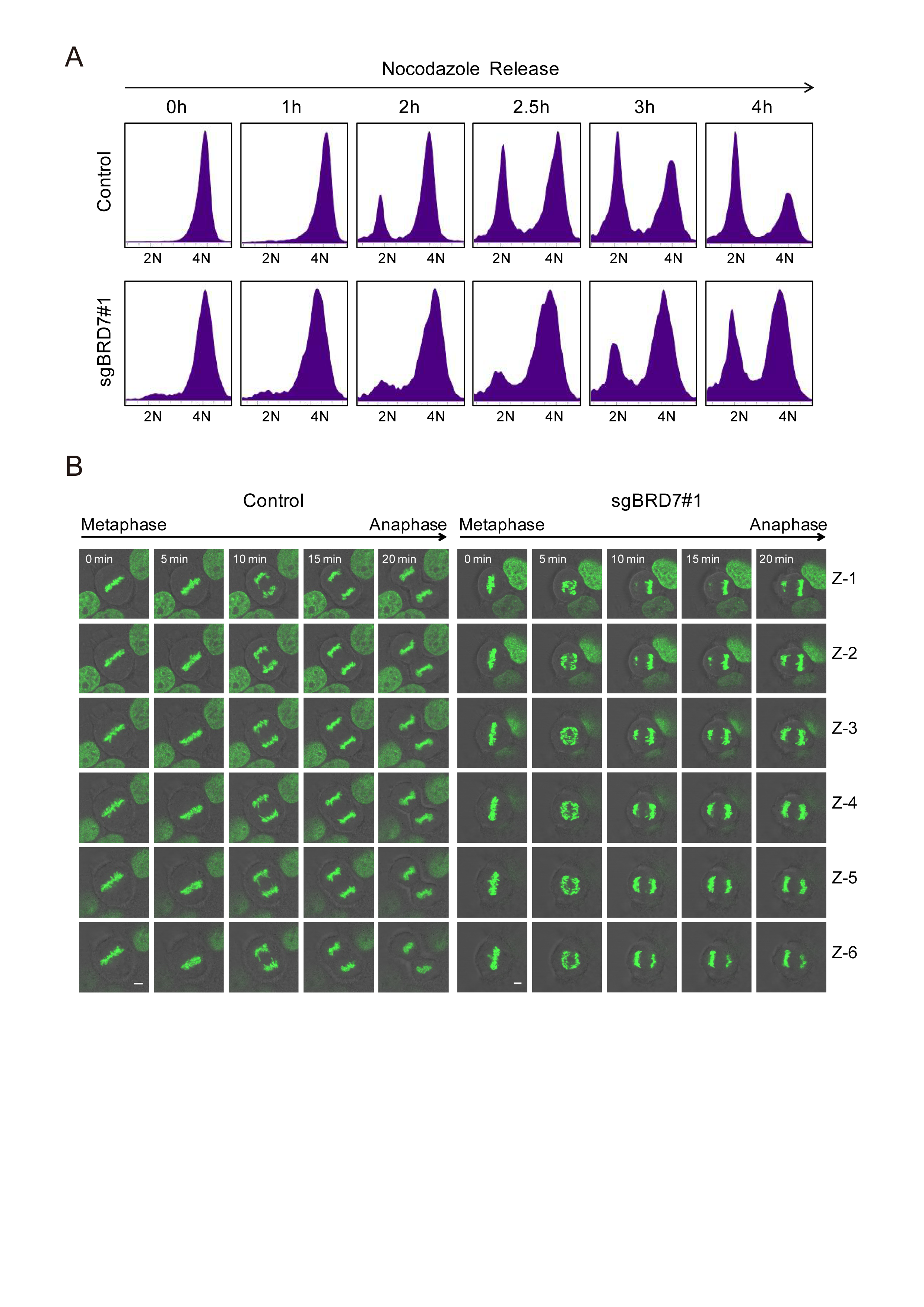


**Supplementary Figure S2. Depletion of BRD7 delays mitotic exit and leads to spindle misorientation in HeLa cells.** A. HeLa cells transfected with indicated sgRNA and synchronized in M phase by incubation with 100 ng/ml nocodazole for 14 h. M phase cells selected by shake-off were released for the indicated time. B. Time-lapse images showing prolonged metaphase to anaphase and misoriented cell division (uneven timing of daughter-cell adhesion to the substratum) in BRD7-depleted HeLa-H2B cells, compared with control. (Scale bars, 2 μm).
